# Muscarinic receptor drug trihexyphenidyl can alter growth of mesenchymal glioblastoma *in vivo*

**DOI:** 10.3389/fphar.2024.1468920

**Published:** 2024-09-25

**Authors:** Renfei Du, Ahmed Y. Sanin, Wenjie Shi, Bing Huang, Ann-Christin Nickel, Andres Vargas-Toscano, Shuran Huo, Thomas Nickl-Jockschat, Claudia A. Dumitru, Wei Hu, Siyu Duan, I. Erol Sandalcioglu, Roland S. Croner, Joshua Alcaniz, Wolfgang Walther, Carsten Berndt, Ulf D. Kahlert

**Affiliations:** ^1^ Chifeng Municipal Hospital, Chifeng, China; ^2^ Clinic for Neurosurgery, Medical Faculty and University Hospital Düsseldorf, Heinrich-Heine University, Düsseldorf, Germany; ^3^ Molecular and Experimental Surgery, Clinic for General-, Visceral -, Vascular- and Transplantation Surgery, Medical Faculty and University Hospital Magdeburg, Otto-von-Guericke University, Magdeburg, Germany; ^4^ FORM, Frankfurt Oral Regenerative Medicine, Clinic for Maxillofacial and Plastic Surgery, Goethe University, Frankfurt Am Main, Germany; ^5^ Department of Cardiothoracic Surgery, First Affiliated Hospital of Shihezi University, Shihezi, China; ^6^ Chifeng Cancer Hospital, Chifeng, China; ^7^ Department of Psychiatry and Psychotherapy, Otto-von-Guericke University, Magdeburg, Germany; ^8^ Clinic for Neurosurgery, Medical Faculty and University Hospital Magdeburg, Otto von Guericke University, Magdeburg, Germany; ^9^ Experimental Pharmacology and Oncology Berlin-Buch GmbH, Berlin, Germany; ^10^ Clinic for Neurology, Medical Faculty and University Hospital Düsseldorf, Heinrich-Heine University, Düsseldorf, Germany

**Keywords:** trihexyphenidyl, glioblastoma, mesenchymal transformation, drug repurposing, cystathionine beta-synthase

## Abstract

Glioblastoma (GBM) is the most commonly occurring and most aggressive primary brain tumor. Transcriptomics-based tumor subtype classification has established the mesenchymal lineage of GBM (MES-GBM) as cancers with particular aggressive behavior and high levels of therapy resistance. Previously it was show that Trihexyphenidyl (THP), a market approved M1 muscarinic receptor-targeting oral drug can suppress proliferation and survival of GBM stem cells from the classical transcriptomic subtype. In a series of *in vitro* experiments, this study confirms the therapeutic potential of THP, by effectively suppressing the growth, proliferation and survival of MES-GBM cells with limited effects on non-tumor cells. Transcriptomic profiling of treated cancer cells identified genes and associated metabolic signaling pathways as possible underlying molecular mechanisms responsible for THP-induced effects. *In vivo* trials of THP in immunocompromised mice carry orthotopic MES-GBMs showed moderate response to the drug. This study further highlights the potential of THP repurposing as an anti-cancer treatment regimen but mode of action and d optimal treatment procedures for *in vivo* regimens need to be investigated further.

## Graphical Abstract

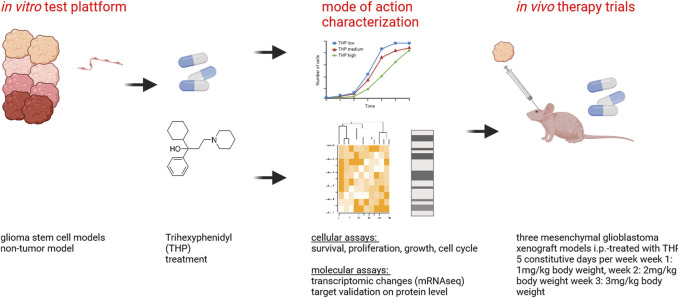

## Introduction

Glioblastoma (GBM) is the most common and highly malignant primary brain tumor. Its highly malignant behavior is characterized by rapid proliferation and invasiveness tumors cells into adjacent brain tissue. Despite radical therapeutic strategies consisting of maximal tumor resection and adjuvant radio-chemotherapy, the median survival time of GBM patients is less than 1.5 years (Mattei et al., 2021).

Molecular diagnostics has revolutionized neuro-oncological diagnostics, particularly since the identification of the IDH1 mutation status, which plays a pivotal role in GBM classification and prognosis. The integration of OMICS technologies has facilitated the molecular sub-classification of gliomas and opened new avenues in personalized neuropathology. Transcriptome profiling revealed the mesenchymal subtype of GBM (MES-GBM) as the most aggressive form of this disease with particularly short overall survival times of the respective patients (Wang et al., 2017). Epithelial-mesenchymal transition (EMT) plays a pivotal role in both the establishment and sustenance of cancer stem cells (CSCs), a specialized cellular population implicated in tumor initiation, recurrence and resistance to conventional therapeutic interventions (Azam et al., 2020). Glioblastoma’s aggressiveness is a consequence of glioblastoma stem cells (GSCs), which exhibit multipotency and self-renewal, similar to neural stem cells (Piccirillo and Vescovi, 2007). Due to their inherent resistance to therapy, GSCs continue to proliferate even after treatment exposure, thereby restoring tumor growth and dissemination. Moreover, undergoing mesenchymal transition, GBM cells enhance their resistance to temozolomide (TMZ), an alkylating chemotherapeutic drug (Kim et al., 2021).

Recent preclinical studies suggest that repurposing existing drugs with cytotoxic and anti-proliferative capabilities could improve the therapeutic options for patients suffering from GBM. As such, we previously identified the therapeutic potential of Trihexyphenidyl (THP, C_20_H_31_NO) in GBM (Vargas-Toscano et al., 2020a). THP is an anticholinergic agent typically used in the treatment of Parkinson’s disease to address symptoms such as tremors, spasms, stiffness and impaired muscle control (Jilani et al., 2024). Considering its only moderate adverse effects, but high permeability in the blood brain barrier (BBB), THP is an outstanding candidate for this investigation (Sogo et al., 2021). THP has been shown to inhibit the proliferation and survival of GSC from the classical GBM transcriptome subtype (CL-GBM). This study aimed to investigate: 1) if THP is able to impair the growth and survival of MES-GBM and 2) if the therapeutic effects can be replicated *in vivo.*

## Materials and methods

### Cell culture

All cell lines were cultured according to a standard protocol in a controlled environment (37°C, 5% CO_2_, humidified). Routine assessments for *mycoplasma* contamination and cell line authenticity were conducted using the short tandem repeat assay, as detailed previously (Kahlert et al., 2015). All cell lines were passaged every other day. In total, five stem cell lines, namely, GBM1 from classical GBM subtype (Aretz et al., 2022); NCH421k (Kološa et al., 2015), JHH520 (Aretz et al., 2022), BTSC233 (Fedele et al., 2017) from mesenchymal subtype and NCH644 from proneural subtype (Podergajs et al., 2013) were used. Commercially available cell lines (U87-GBM and HUVEC) were used as control. HUVECs were purchased from PromoCell GmbH Heidelberg, U87 was kindly provided by A Weyerbrock, formerly at Neurosurgery Department Medical Center Freiburg, Germany. All sources and specific media components are detailed in Supplementary Tables S1–S4.

### Cell viability assays

The CellTiter-Glo^®^ (CTG) solution (Promega, Madison, WI) was prepared according to manufacturer description. The resulting THP was initially resuspended to a 50 mM concentration in methanol (MeOH) and subsequently diluted to the required concentrations for the experiment. Cell lines were dissociated using TrypLE, washed then adjusted to a density of 2000 cells in 100 µL of complete media per well. Cells were allocated into black 96-well plates in technical triplicates, with THP concentrations set at 10, 20, 30, 40, and 50 μM, alongside a MeOH control, all maintained in biological triplicates. Cells were subsequently incubated with THP for durations of 0, 2, 4, and 6 days under standard culture conditions (humidified atmosphere, 37°C, 5% CO_2_). Plates were retrieved at specified time points (0, 2, 4, and 6 days) followed by the luminescence development. Cell viability was quantified using micro plate reader (Tecan, Austria).

### Cell proliferation assessment

Cell proliferation levels were assessed by Ki67 expression allowing quantification of the percentage of proliferating or non-proliferating cells. Proliferation of tumor cells with or without THP treatment was analyzed after 48 and 72 h using the Muse Ki67 Proliferation Kit. Briefly, after routine centrifugation and washing, an osmotic stabilization solution was added (1 × 10^5^ cells to a 1.5 mL tube), followed by the addition of Muse IgG1-PE or Muse Hu Ki67 PE antibodies. The Muse^®^ cell analyzer was used to perform cell sorting analysis. Results were expressed as the percentage of Ki67^+^ cells and Ki67^-^ cells.

### Cell cycle analysis

Cell cycle analysis was conducted on all cell lines following 48 and 72 h of incubation with and without a 10 µM concentration of THP. Each cell line was processed in triplicate. For the analysis, tumor cells were fluorescence stained using the Muse^®^ Cell Cycle kit (Guava Muse^®^, USA) and analyzed with the Muse cell analyzer. In brief, the supernatant was removed by routine centrifugation (1 × 10^5^ cells to a 1.5 mL tube), the cells were mixed and ice-cold 70% ethanol was added. 200 μL of Cell Dispersion Reagent was added and left in the dark for 30 min at room temperature. For each sample, a total of 1 × 10^4^ events were recorded. The data were analyzed using the Muse^®^ Cell Cycle software module, facilitated the calculation of cell cycle distributions. The number of gated cells in the G0/G1, S, and G2/M phases were quantified and expressed as percentages.

### Apoptosis assay

Fluorescence detection of annexin V served to evaluate the apoptotic events induced by THP treatment for 48 and 72 h. Expression in THP-treated or untreated cells was examined using the Muse^®^ Annexin V & Dead Cell Kit. The supernatant was removed by routine centrifugation (1 × 10^6^ cells to a 1.5 mL tube), treated with 500 µL TrypLE for 3 min, rinsed the cells and 100 µL of detection reagent was added, incubated in the dark at room temperature for 30 min. Thereafter, stained cells were subjected to Muse^®^ cell analyzer. Muse^®^ cell software was served to calculate the percentage of early and late apoptotic cells, as well as necrotic and vital cells.

### RT-qPCR

Total RNA was extracted from cultured cells using TRIzol reagent (Invitrogen) (1,000,000 cells), followed by reverse transcription to synthesize cDNA. Quantitative real-time PCR (qRT-PCR) was performed using BioRad CFX Connect Real-Time System qPCR cycler. Primers, synthesized by Sangon, targeted specific genes, and relative mRNA expression levels were calculated using the method. Primer sequences of HIF-2α, CBS and ZEB1 was detailed in Supplementary Table S5.

### mRNA-sequencing analyses

THP-treated or untreated cells (GBM1, NCH644, JHH520) were subjected to RNA sequencing after 48 h. Total RNA samples designated for transcriptome analyses were quantified using the Qubit RNA HS Assay (Thermo Fisher Scientific) and assessed for quality via capillary electrophoresis with the FragmentAnalyzer and DNF-471 Standard Sensitivity Assay (Agilent Technologies, Inc., Santa Clara, USA). All samples achieved excellent RNA Quality Numbers (RIN >8.8). Library preparation was carried out according to the ‘VAHTS Universal V6 RNA-Seq Library Prep Kit for Illumina V9.1’ protocol. Briefly, 400 ng of total RNA was employed for mRNA capture, fragmentation, cDNA synthesis, adapter ligation, and library amplification. Following bead purification, libraries were normalized and sequenced on the HiSeq 3,000 system (Illumina Inc., San Diego, USA) using a 2 × 150 bp read setup. The bcl2fastq2 software (version 2.17.1.4) was utilized to convert bcl files into fastq files, and to perform adapter trimming and demultiplexing.

All raw data were processed by R software with the screening criteria of logarithmic fold change |log FC|>1.5 and *p* < 0.05. Afterwards, Venn diagram was used to identify the shared differentially expressed genes in the three datasets. Finally, Gene set enrichment analysis (GSEA) was performed using Kyoto Encyclopedia of Genes and Genomes (KEGG) pathway enrichment analysis identified the signaling pathways significantly enriched by the feature (Minoru et al., 2021). The complete KEGG gene set list was downloaded from mSigDB database https://www.gsea-msigdb.org/gsea/msigdb(accessed autumn 2023).

The survival analysis of genes of interest were executed on data from The Cancer Genome Atlas (TCGA) using TCGAbiolinks R package and statistical testing was performed using log rank test.

### Western blot-based protein analysis

Western blot was used to evaluate the CBS related protein expression of the THP processed to each tumor cell line (GBM1, NCH644, JHH520) after 72 h. Additionally, the changes in protein expression after 48 and 72 h of treatment were observed in JHH520. Total proteins were isolated using RIPA buffer (1 × 10^5^ cells to a 1.5 mL tube). Tumor cell lysates were resolved on ready-made BAA gels, with the gel concentration selected based on the molecular weight of the target proteins. Electrophoresis was conducted for 60 min at 110 V. Proteins were then transferred to nitrocellulose membranes over a period of 1 hour at the same voltage. The membranes were subsequently blocked with nonfat dry milk for 1 h to prevent nonspecific binding, followed with the primary antibodies and secondary antibodies were applied. Proteins were finally identified by chemiluminescence (Bio-Rad, USA).

### Oxyblot analysis

Protein oxidation after THP treatment was analyzed using the OxyBlot™ Protein Oxidation Detection Kit (S7150, Merck Millipore). Briefly, protein samples (5 ng/μL for all samples) were resuspended in 6% SDS buffer and subsequently derivatized with or without 2,4-dinitrophenylhydrazine. Equal amounts of derivatized and non-derivatized samples (2.5 μg/sample) were resolved by SDS-PAGE, transferred from a polyacrylamide gel to the membrane. Thereafter, membranes were incubated with the first antibody (90,450) (1:150 in 1% bovine serum albumin/PBS) for 1 h at 25°C, followed by the secondary antibody (90,451) (1: 300 in 1% bovine serum albumin/PBS) for 1 h at 25°C. The oxidatively modified proteins were finally detected by SuperSignal West Pico chemiluminescent substrate (Pierce Biotechnology). Band intensity data were obtained using ImageQuant TL software (GE Healthcare).

In addition, we detected oxidized and persulfidated proteins using the protein persulfide detection protocol (ProPerDP) established by Dóka et al. (2016).

### Animal model generation

Animal studies were performed in accordance with the German Animal Welfare Act and approved by local authorities (Landesamt für Gesundheit und Soziales, LaGeSo Berlin, Germany) under the permission E0023-23. 6–8 weeks old NMRI nu:nu mice (Janvier Labs) were used. Intracerebral injections were performed as previously described (Alcaniz et al., 2023). Prior to surgery, all mice received 4 mg/kg meloxicam subcutaneously and were anesthetized with a combination of 90 mg/kg ketamine and 12 mg/kg xylazine. Anesthetized mice were fixed in a stereotactic frame, the skin on the skull was opened and 2 μL cell suspension containing 1 × 10^5^ cells were slowly injected through a hole in the skull into the cortex of the right hemisphere. After cell injection, the syringe was removed slowly and the skin was closed using tissue adhesive. The following day mice received 4 mg/kg meloxicam subcutaneously again.

### Therapeutic trials

Treatment with THP started on day 12 after tumor cell inoculation. Animals received THP as i.p. administered monotherapy five constitutive days per week starting with first week dosage 1 mg/kg body weight, week 2 with 2 mg/kg body weight and week 3 with 3 mg/kg body weight. Bioluminescence imaging was performed once per week with the NightOwl II LB983 *in vivo* imaging system. The IndiGO 2.0.5.0 software is used for initial analysis, color-coding of the signal intensity and quantification. Animal condition was checked daily and body weight was measured at least twice weekly. Mice were sacrificed for ethical reasons when showing behavioral abnormalities and body weight loss, surrogates for progressive intracranial tumor growth.

### Statistics

All *in vitro* experiments were performed with a minimum number of three biological repetitions, each in three technical replicates. Statistical significance was calculated with ANOVA or a *t*-test. All statistical calculations and graphing were done through GraphPad Prism 8.0 software (La Jolla, USA). Significant findings (*) with a *p*-value <0.05 and highly significant findings (**) with a *p*-value <0.01 or (***) with a *p*-value <0.001. All statistical calculations and graphing were done through GraphPad Prism 8.0 software (La Jolla, USA) and R soft (Version 4.2.0).

## Results

### THP can inhibit growth of GBM stem cells from different tumor subtypes with limited effects on human vascular endothelial cells

**FIGURE 1:**
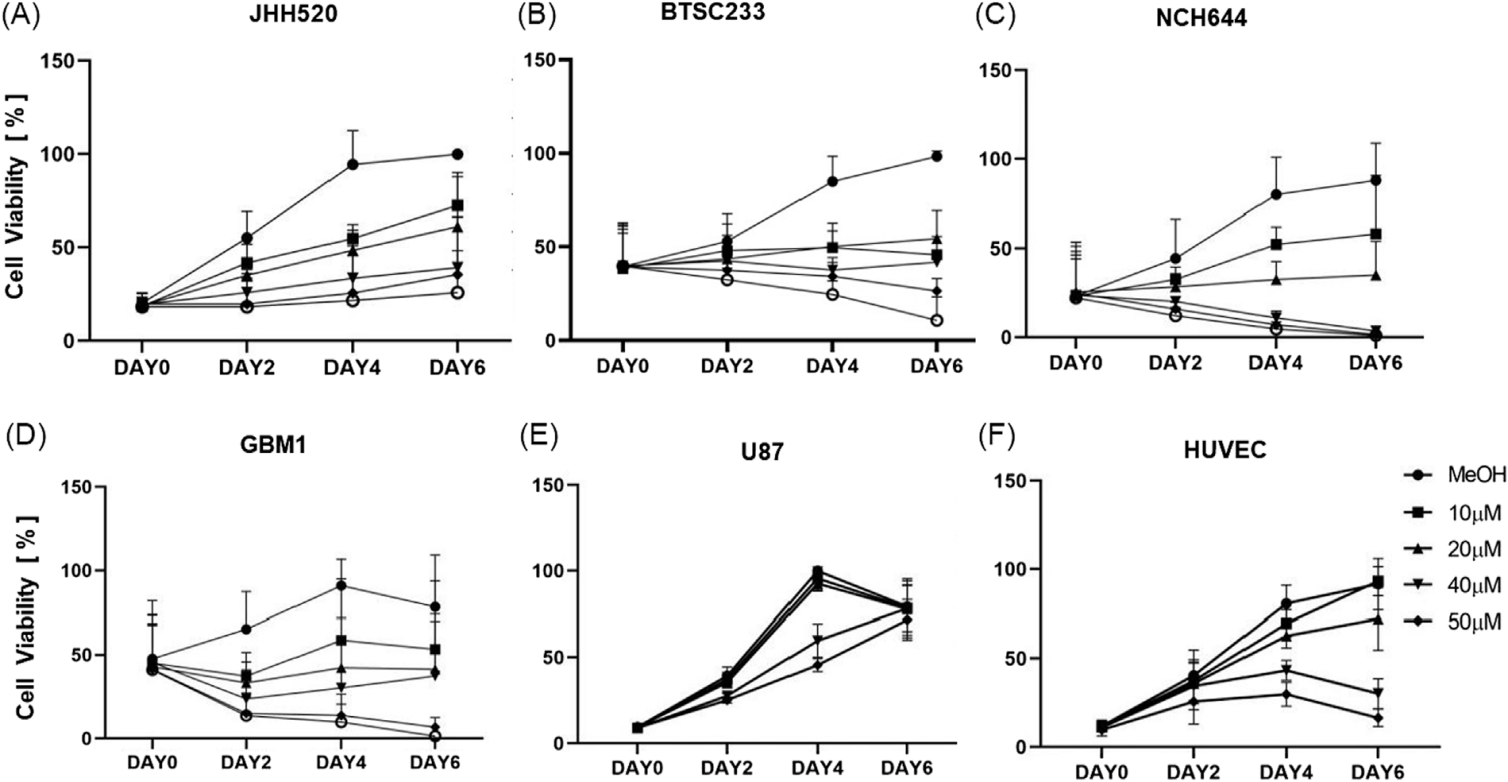
Cell viability of all the cell lines (JHH520, BTSC233, NCH644, GBM1, U87 and HUVEC) using ATP consumption assay treated with five different concentrations of THP. Control samples were treated by MeOH without THP. Each experiment executed at least n = 3 independent repetitions **(A–F)**.

THP therapeutic potential was replicated and confirmed in all tested models, independently of the molecular subclass of glioblastoma they represent. The efficacy of varying concentrations of THP on tumor stem cell models, as well as on the non-cancerous control cell line HUVEC, was assessed using the CTG assay at intervals of 0, 2, 4, and 6 days. Overall, THP treatment led to a significant reduction in the viability of tumor stem cells in a dose-dependent manner, as illustrated in Figure 1. Of note, classical monolayer cultured U87 grown in serum containing medium did show relative high levels of resistance to the drug, revealing growth reduction on only on day 4 under high dosage therapy. This dose-response relationship became increasingly evident over time. At a concentration of 10 μM, THP’s impact on HUVEC cells was minimal. Interestingly, on day 6, the viability of HUVEC cells treated with 10 µM THP surpassed that of the untreated control group, suggesting that THP exhibits low toxicity towards non-malignant cells at this concentration. Based on these results, we selected the optimal concentration of 10 µM THP for subsequent experiments. These findings indicate that THP could offer a potent therapeutic advantage in glioblastoma treatment, with reduced adverse effects on healthy cells (Figure 1).

### THP treatment can inhibit proliferation of GBM stem cells with limited effects on human vascular endothelial cells

**FIGURE 2:**
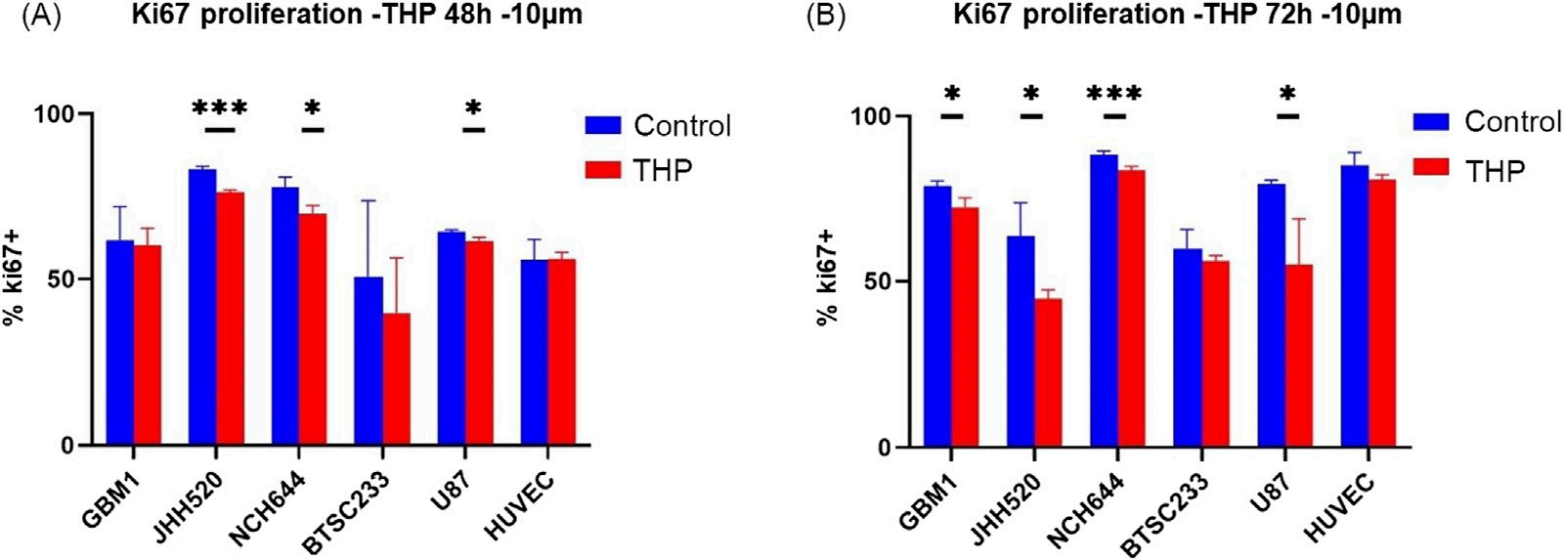
Proliferation results using FACS based quantification for 10 µM THP treatment of cells for 48 and 72 h. Cell proliferation was expressed as percentage of Ki67-positive cells to total amount of gated events recorded. Control samples were without THP treatment **(A,B)**. Each experiment executed at least for 3 independent repetitions. Significant findings (*) with a *p* value < 0.05 and highly significant findings (**) with a *p* value < 0.01 or (***) with a *p* value < 0.001.

Subsequent assays were conducted on cells treated with 10 µM THP concentration for 48 h and 72 h. Analysis of the Ki67 proliferation index revealed significant differences in three out of five GBM cell lines (Figure 2). Specifically, the proliferation rates of JHH520, NCH644, and U87 were significantly reduced after 48 h of THP treatment. In contrast, GBM1 and BTSC233 did not show significant differences in their proliferation rate, and neither did the control cell line HUVEC. At 72 h post-treatment, all cell lines, with the exception of BTSC233 and the non-cancerous HUVEC cells, demonstrated a significant reduction in proliferation. These results suggest that a concentration of 10 µM THP effectively inhibits the proliferation of a majority of glioma stem cells, while exerting negligible effects on non-malignant cells.

### THP treatment does not affect cell cycle progression of GBM stem cells

**FIGURE 3:**
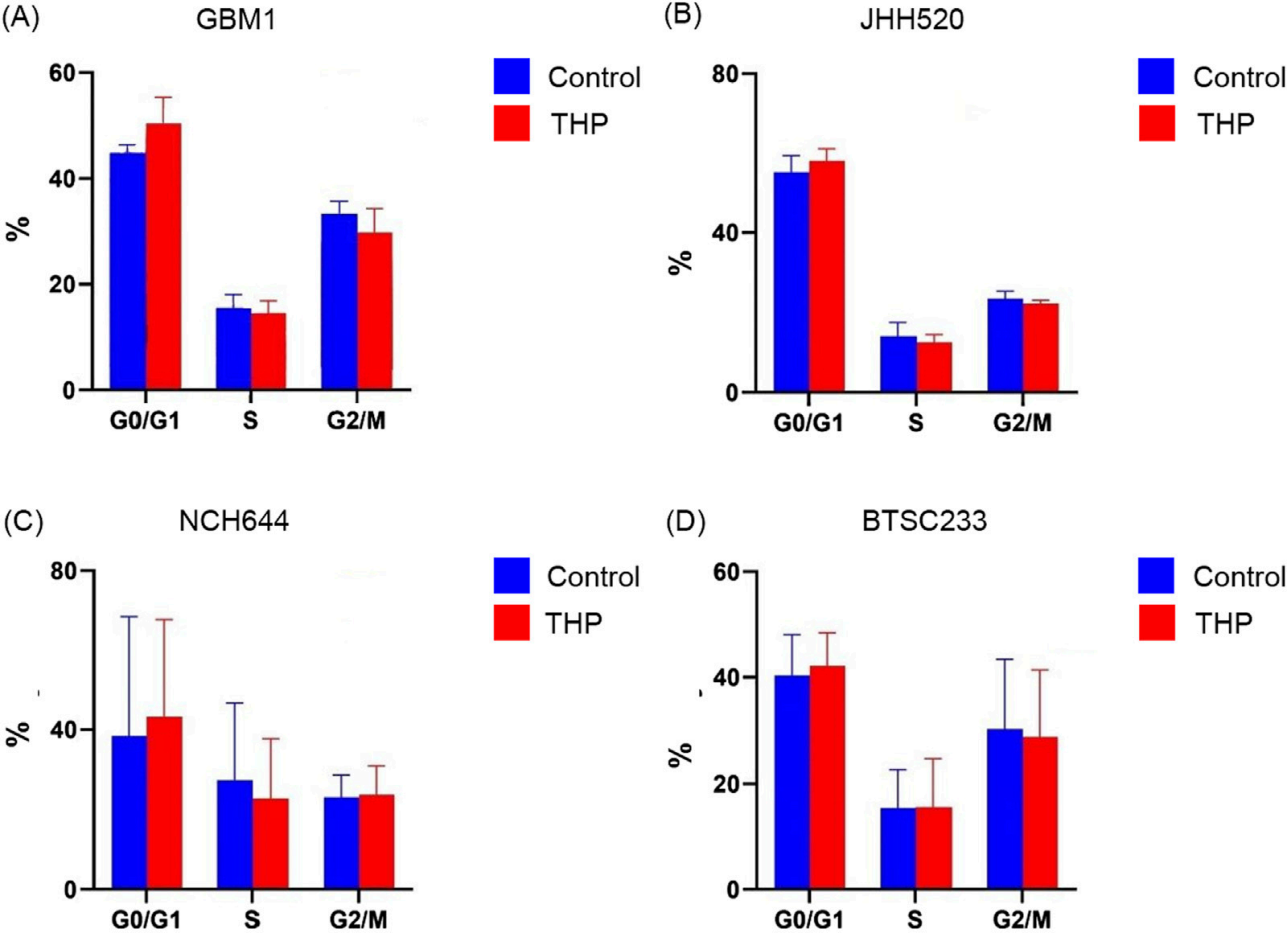
Cell cycle distribution in GBM1, JHH520, NCH644 and BTSC233 cells following 10 µM THP treatment for 48 h. Control samples were without THP treatment. Each experiment executed at least for 3 independent repetitions **(A–D)**.

To evaluate the impact of THP on the cell cycle, we treated the stem cell models with 10 µM THP for 48 h. The results showed no significant cell cycle alterations in any of the cell lines, although a slight increase in the G0/G1 phase upon THP treatment was detected (Figure 3). These findings indicate that the inhibitory effects of THP on the growth and proliferation of glioma stem cells are most likely not a consequence of cell cycle alterations. Although all our experiments are performed under standardized conditions as defined by our previously described quality control system (Hewera et al., 2020) enforcing highly repetitiveness of genetic and functional properties of our *in vitro* platform (Nickel et al., 2021) we acknowledge the fluctuate growth of cell model NCH644 in between the different biological repetitions. This results in large error bars when evaluating the cumulative data consequently also impacting the statistical results.

### THP treatment caused significant induction of apoptosis in GBM stem cells with limited effects on human vascular endothelial cells

**FIGURE 4:**
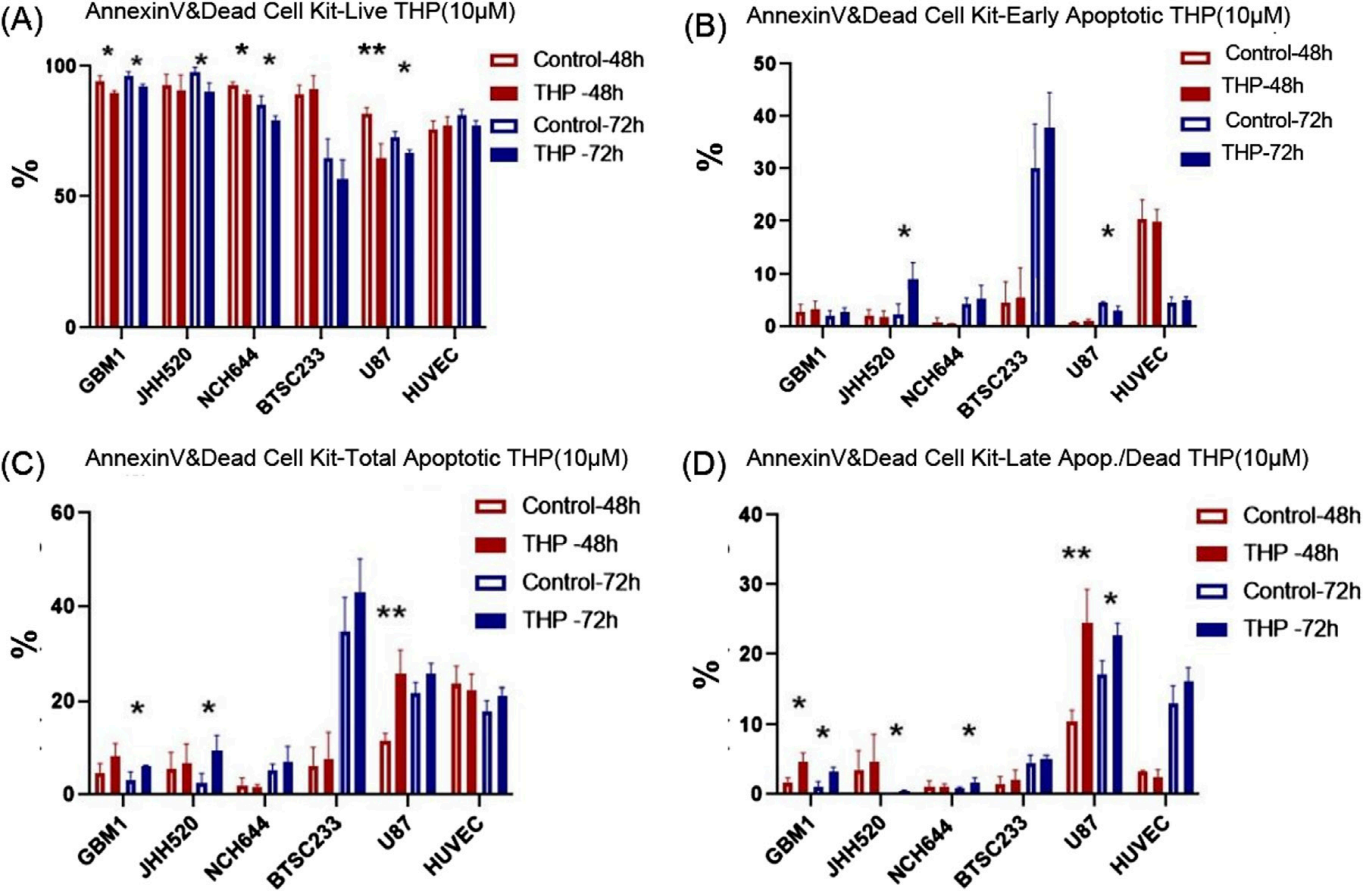
Apoptosis analysis results using Annexin V/PI staining and FACS based quantification for 10 µM THP treatment of cells for 48 and 72 h. Viable cells **(A)**, early apoptotic cells **(B)**, total apoptotic cells **(C)** and late apoptotic cells **(D)**. Control samples were without THP treatment. Each experiment executed at least for three independent repetitions. Significant findings (*) with a *p*-value <0.05 and highly significant findings (**) with a *p*-value <0.01 or (***) with a *p*-value <0.001.

To ascertain whether THP’s cell-killing effect was mediated through apoptosis, all cell lines were treated with 10 µM THP for 48 and 72 h, followed by Annexin-V staining to detect apoptotic cells. The results showed that NCH644, GBM1, and HUVEC cell lines did not undergo apoptosis at neither time point, with only minimal differences in cell viability observed. In contrast, U87 cells exhibited a significant increase in apoptosis after 48 h (*p* < 0.01), a trend that persisted at 72 h. Interestingly, the most notable apoptotic response to THP treatment was observed in the BTSC233 cell line, where the percentage of apoptotic cells significantly increased at 72 h post-treatment (Figure 4). Together with the previous observations, these results suggest that THP’s inhibitory action on certain glioma stem cell types could be attributed to its ability to induce apoptosis, although our data focused on assessing late and early apoptosis only.

### THP induces transcriptional upregulation of CBSL and other metabolic genes in GBM stem cells

**FIGURE 5:**
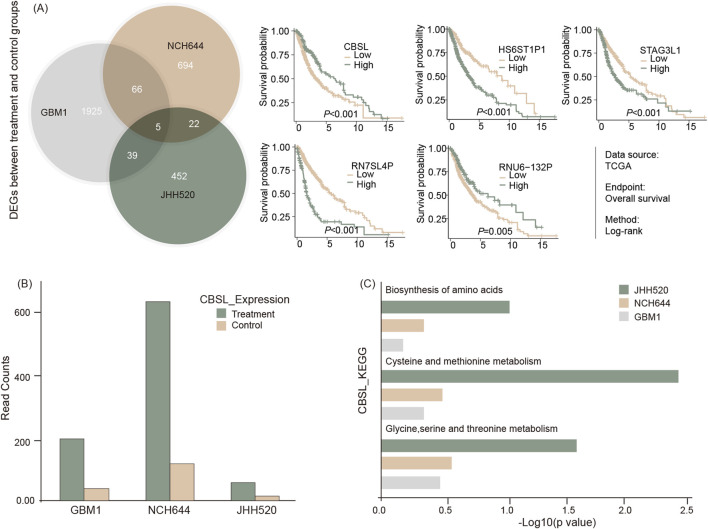
RNA sequencing of cell lines with or without THP treatment for 48 h. Shared regulatory genes were processed and represented via Venn diagram and subsequent survival analysis of five identified gene signals using TSCG dataset **(A)**, differential expression of CBS-L **(B)**, and GSEA result by KEGG for the related pathways **(C)**.

To investigate the molecular impact of THP on GSCs, total mRNA sequencing was conducted on three GBM stem cell lines: GBM1, JHH520, and NCH644. The cells were treated with 10 µM THP for 48 h, followed by RNA extraction and RNA sequencing. The results showed that that only five genes were differentially regulated across all 3 cell lines upon THP treatment. Four of these were pseudogenes, which are coding gene copies lacking normal function in protein coding (HS6ST1P1, RN7SL4P, RNU6-132P, STAG3L1). The fifth gene, Cystathionine beta-synthase-like (CBS-L), showed significant upregulation in all 3 cell lines (Figures 5A, B). CBS-L is involved in the transsulfuration pathway, crucial for metabolizing L-methionine and detoxifying L-homocysteine.

In further studies, the mRNA sequencing data from THP treated versus non-treated cells was subjected to KEGG pathway analyses. The results showed that the pathways associated with amino acid biosynthesis and the metabolism of cysteine, methionine, glycine, serine, and threonine were upregulated in the THP-treated samples in all cell lines (Figure 5C). These findings suggest that the effect of THP on GBM cells may occur via alterations of the molecular pathways associated with cell metabolism.

### CBS-L protein analysis and quantification of reduced, oxidized, and persulfidated cysteines

**FIGURE 6:**
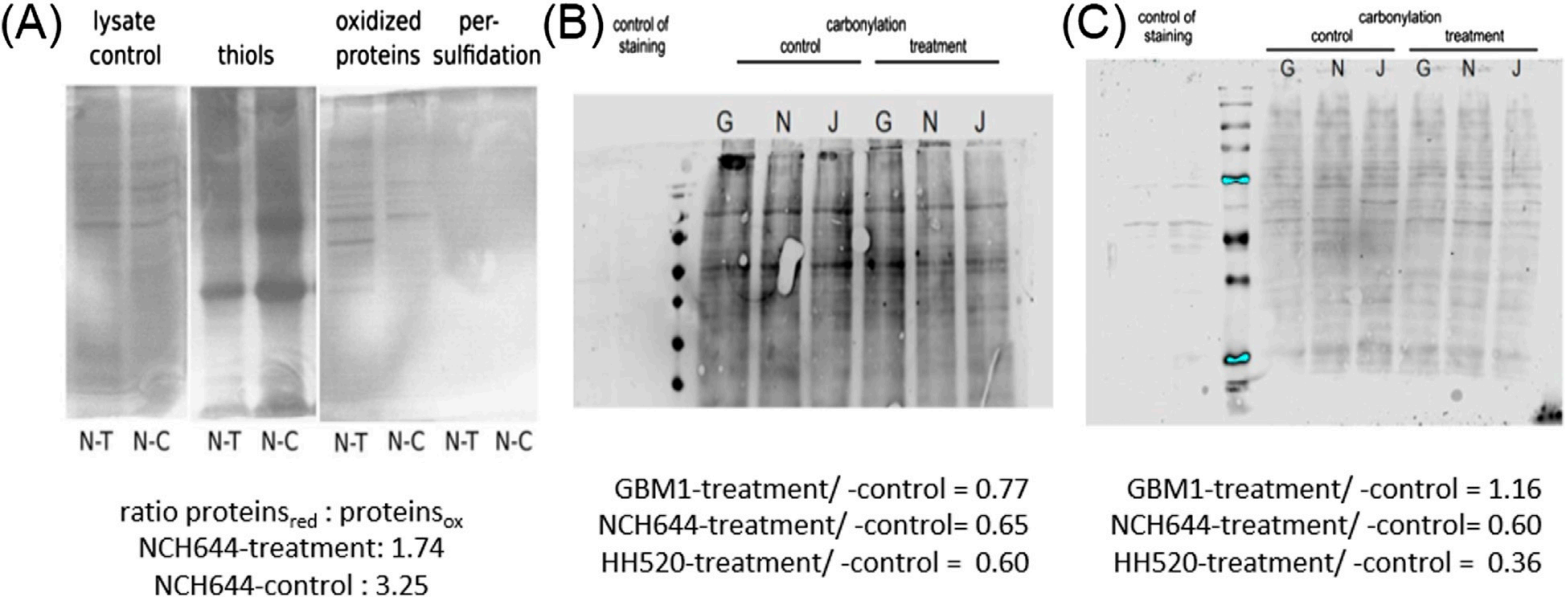
Detection of persulfidated proteins in NCH644 48 h post THP treatment **(A)**. Oxyblot THP 48 h -GBM1/NCH644/JHH520 **(B)**. Oxyblot THP 72 h -GBM1/NCH644/JHH520 **(C)**.

Western blot analyses were conducted to confirm the impact of THP on CBS-L protein levels in three tumor cell lines (GBM1, JHH520, and NCH644) treated with 10 µM THP for 72 h. However, CBS-L is not detected by anti-CBS antibodies, or the effect on mRNA is not visible on protein level. Despite observing various dysregulation in all 3 cell lines, the changes were not statistically significant (Supplementary Figure S1A). Treatment of cell line NCH644 for 48 h showed again no significant differences compared to the control group (Supplementary Figure S1B). To further explore the assumed role of CBSL in H_2_S formation during THP treatment, we detected protein persulfidation, the oxidative posttranslational modification of cysteine residues initiated by H_2_S. Unfortunately, were not able to see persulfidated proteins. However, the ratio of oxidized to reduced proteins nearly doubled following THP treatment whereas the number of protein thiols (reduced cysteine residues) decreased, indicating significant changes in the oxidative state of proteins (Figure 6A). Further analysis was conducted to assess protein carbonylation in three GBM cell lines at 48 h and 72 h post-THP treatment (Figures 6B, C). In line with previous findings demonstrating that H_2_S diminished protein carbonylation (Koike et al., 2016; Mao et al., 2022), protein carbonylation levels decreased in NCH644 and JHH520 cells at both time points. However, the GBM1 cell line exhibited a slight increase in carbonylated proteins after 72 h of THP treatment, suggesting variable responses to THP across different GBM cell lines. These findings underscore the complex effects of THP on protein chemistry within tumor cells.

### THP alters the growth of mesenchymal GBM *in vivo*

**FIGURE 7:**
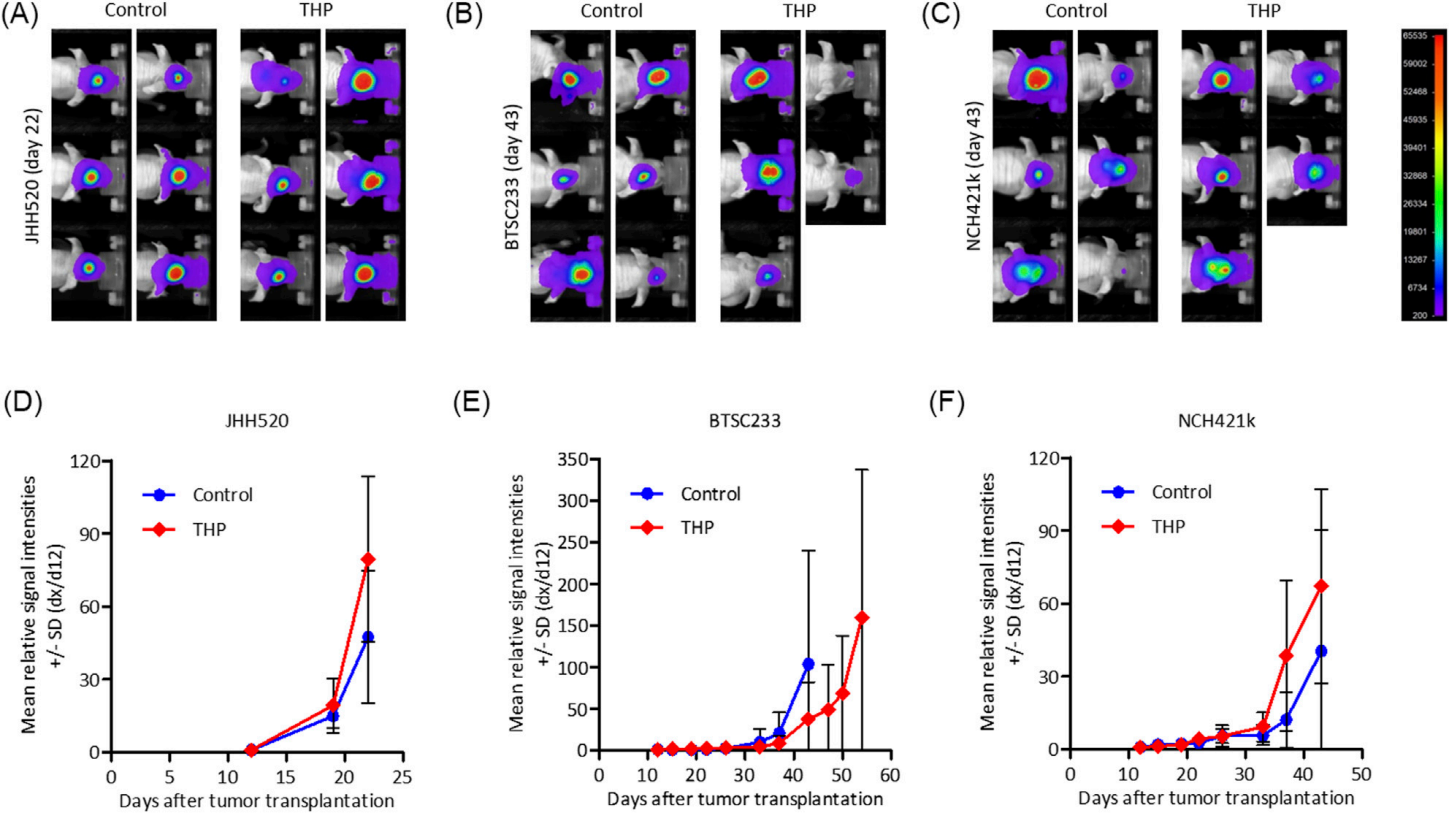
Bioluminescence based assessment of orthotopic tumor growth in immunocompromised mice for mesenchymal tumor subtype cell models BTC233, NCH421k and JHH520. **(A–C)** Exemplary images of animals in treatment groups at latest time point when both groups were still complete. **(D–F)** Mean relative signal intensities (day x to day 12) until measurement when individual groups were still complete. n = 5 or 6.

Three PDX models of MES-GBM were established and treated with THP as described. JHH520 showed the highest *in vivo* tumor formation capacity and comparably homogeneous intracranial growth, Models BTSC233 and NCH421k grew approximately half as fast (Figure 7). In models JHH520 and NCH421k we did not observe tumor growth inhibition, with signal intensities under treatment with THP increasing comparably to control groups, albeit being slightly higher on average. Furthermore, THP had no effect on surrogate survival in these two models. First animals in THP groups and their respective control groups had to be terminated for ethical reasons within 2 days. In model BTSC233 mean signal intensities increased comparably in control animals and animals treated with THP for the first 2 weeks, but eventually decreased in individual animals in the THP group in treatment week three. Furthermore, first two animals in the control group had to be terminated on day 43, while first animals treated with THP had to be terminated on day 54. While monitored differences were not significant, this overall indicates some degree of tumor growth inhibition of THP in the orthotopic BTSC233 *in vivo* model. THP did not cause any side effects in the performed studies.

## Discussion

Utilizing a variety of GSC models *in vivo* and *in vitro* featuring three model systems of MES-GBM subtype, this project revealed the anti-neoplastic potential of THP. *In vitro* experiments using HUVEC cells suggest that the drug exposes minimal effects on non-cancer cells, but further validation with model system featuring non-cancer cells of the brain are needed to verify this hypothesis. mRNA sequencing analysis of treated GSCs identified a dysregulation of CBS-L transcription as one possible mediator of the observed cellular responses *in vitro*.

THP, a commonly used anticholinergic drug for treating Parkinson’s disease, is typically administered to young and cognitively normal patients (Nabors et al., 2014). Recently, our team identified THP as a promising candidate for glioma treatment, as indicated by its efficacy in a case report (Vargas-Toscano et al., 2020a). Notably, THP is well-tolerated in humans at high doses and can penetrate the blood brain barrier, which makes it a suitable candidate for clinical application (Brocks, 1999). The results presented in this study proved the inhibitory effect of the drug i, and expanded the range of *in vitro* and *in vivo* cell models to validate our findings. Our research provides new insights to the field, in particular that THP exhibits minimal effects on non-cancerous vascular cells, suggesting a higher therapeutic index for rapidly proliferating neoplastic cells compared to normal cells. This implies a lower risk of off-target effects in tumor treatments. Additionally, THP *in vitro* has proven effective against GBM cells with diverse molecular properties, including the most aggressive subtype of mesenchymal GBM (Verhaak et al., 2010) and in tumor models with different DNA mutation profiles as previously characterized (Nickel et al., 2021), indicating a broad anti-glioma potential for this drug. Particularly noteworthy is the induction of apoptosis in the stem cell models of the disease, which are generally resistant to standard of care (SoC) treatment such as TMZ and ionizing radiation (IR) therapy (Vargas-Toscano et al., 2020b; Suwala et al., 2018). A comparative assessment of monotherapy THP and THP in combination treatment with TMZ + IR is needed to hypothesize any additive effect of our approach to clinical SoC.

In first *in vivo* experiments we observed activity of THP in one out of the three tested cell-line derived xenograft (CDX) models. THP delayed orthotopic BTSC233 tumor growth, as seen in the lower bioluminescence intensities and the indicated longer survival of individual animals. Interestingly, effects here were visible from treatment week three on, when animals started to receive the highest dose of THP. This suggests that an optimized treatment regimen with a higher THP dose from study start on might be beneficial. Whether this can further improve treatment outcome in the BTSC233 model and lead to tumor growth inhibition in the other orthotopic *in vivo* models JHH520 and NCH421k, both sensitive towards THP *in vitro*, needs further investigation.

The *In vivo* responder models are highly activated in PI3K signaling. As such, BTSC233 is known to express PIK3CA mutation (Nickel et al., 2021), encoding for a subunit for phosphatidylinositol 3-kinase. NCH421k cells are responding with induction of cell death when pharmacologically inhibiting PIK3 signaling (Pareja et al., 2014). JHH520 does not carry PIK3-pathway activating mutations, as assessed via sequencing hotspot loci in PIK3R1 or PIK3CA (Nickel et al., 2021). Comparative analysis of the xenografts extracted from mice treated with THP versus vehicle control is required to test for the relationship between PI3K activation and the mutation status of genes involved in the signal transduction within the individual tumor cells’ response to therapy *in vivo*. With this discrepancy of *in vitro* and *in vivo* THP treatment response data of the same human cancer model, our results exemplify the importance of including disease models representing tumor microenvironment alongside tumor cell parenchyma in projects dedicated to drug/therapy testing.

RNA sequencing results of *in vitro* treated cells demonstrated the upregulation of CBS-L in THP-treated GSCs across all 3 cell lines tested. CBS is involved in the transsulfuration pathway and is essential for the metabolism of L-methionine and the detoxification of L-homocysteine (Perreault et al., 2024). However, subsequent protein level assays yielded different results across the cell lines, possibly due to the longer protein half-life under normoxic conditions or that CBSL is not detected by the applied anti-CBS-antibodies. Further downstream assessments to investigate possibly altered cell metabolic properties of GBM upon treatment with THP are required to identify the cellular function of CBSL and to verify any involvement of CBSL in the observed mode of actions of THP’s anti-cancer effect(s). Moreover, the used sequencing method cannot completely cover the entire transcriptome such as whole exon sequencing, thereby we can not exclude transcriptional level targets of THP to remain undetected in our dataset. Interestingly, TCGA-data based assessment of prognostic value of CBSL expression revealed increased expression correlates with significantly prolonged median overall survival of GBM patients. In relations, Takano et al. (2014) showed that increasing the expression of CBS diminishes the stem cell properties of GBM cells. Taken together, we speculate that a transcriptional activation of CBSL in GBM cells due to treatment with THP decreases tumors malignancy.

THP has demonstrated effectiveness against GSCs. Whether this is via targeting the neurotransmitter pathway connecting glioma cells remains to be investigated. Muscarinic acetylcholine (mACh), acetylcholine (ACh) receptors as well as monoamine pathways related to serotonin (5-HT), dopamine, and norepinephrine are modulated by THP (Thompson and Sontheimer, 2019; Jiang et al., 2020), evidently showing promising therapeutic effects in glioblastoma. The present study aligns with recent state-of-the-art studies on neurotransmitter biology in the context of GBM progression (Vargas-Toscano et al., 2020a; Venkataramani et al., 2019). THP might be an effective approach to disrupt neuron-to-cancer cell synaptic connections. Dedicated studies on disease models representing the neuronal tumor microenvironment of GBM, ideally of full human nature, are required to verify our hypothesis.

THP can inhibit the growth of MES-GBM. Given the good safety profile of the drug, its rather low price and its ability to cross the blood brain barrier, further research to expand on our results would contribute to the development of improved therapeutic strategies for patients with GBM. THP is well tolerated in humans when administered with up to 120 mg per day (Robottom et al., 2011), in increasing dosage regime. This treatment equalizes a daily dosage of roughly 2 mg/kg (70 kg human body weight), therefore justifying the translatability of our selected animal treatment regime into human setting. However. our data shows certain limitation of direct clinical relevance of THP therapy for glioblastoma patients as *in vivo* growth reduction was moderate at best. Speculatively, the potential of THP as anti-GBM drug in combination with standard of care therapy is needed to evaluate the clinical relevance of these findings. Optimizing the treatment regimes, such as sequential combination therapy of chemotherapy and THP or intracranial administration of THP into CNS will benchmark the clinical potential more comprehensively as this study has done. Of note, our data shows that intra-class variations of response of MES GBM to THP exists featuring JHH520 as low responder model. The conducted experiments are unable to reveal underlying genetic properties of GBM cells or molecular networks associated to differences in THP response. Identification of biomarkers allowing the stratification of GBM sensitivity to TMZ might be helpful to increase the overall clinical potential of THP therapy. Follow up studies are needed to address this possibility.

## Data Availability

The datasets presented in this study can be found in online repositories. The names of the repository/repositories and accession number(s) can be found in the article/Supplementary Material.
