# Supplementary material for: Muscarinic receptor drug trihexyphenidyl can alter growth of mesenchymal glioblastoma in vivo
Source: Front Pharmacol. 2024 Sep 25;15:1468920. doi: 10.3389/fphar.2024.1468920 (PMC11461351; doi:10.3389/fphar.2024.1468920)
Supplement: Supplementary file 1 [file DataSheet1.docx]

Supplementary Table S1. Cell line source and growth properties

| Cell line | Origin |
| --- | --- |
| GBM1 (Suspension) | A. Vescovi, San Raffaele Hospital, Milano, Italy |
| NCH644 (Suspension) | C. Herold-Mende, Heidelberg University, Heidelberg, Germany |
| BTSC233 (Suspension) | M.S. Carro, Freiburg University, Freiburg im Breisgau, Germany |
| JHH520 (Suspension) | G. Riggins, Johns Hopkins, Baltimore, MD, USA |
| NCH421k (Suspension) | C. Herold-Mende, Heidelberg University, Heidelberg, Germany |
| U87 (Adherent) | ATCC, No.: CVCL_0022 |
| HUVEC (Adherent) | Promocell, No.: C-22010 |

Supplementary Table S2. Universal cell culture medium for GBM1, NCH644, BTSC233 and JHH520

| Name | Manufacturer | Amount |
| --- | --- | --- |
| DMEM, (+) 4.5g/L D-glucose, (-) Pyruvate | Thermo Fisher, 41965-039 | 340 mL |
| F-12 supplement | Thermo Fisher, 21765-029 | 150 mL |
| B27 Supplement | Thermo Fisher, 17504001 | 10 mL |
| Human EGF | Peprotech, AF-100-15-1mg | 500µL |
| FGF | Peprotech, 100-18B-1mg | 500µL |
| Heparin | Sigma, H0878-100KU | 500µL |
| Penicillin-Streptomycin | Sigma, P4333-100 mL | 5 mL |

Supplementary Table S3. Cell culture medium for U87

| Name | Manufacturer | Amount |
| --- | --- | --- |
| DMEM, (+) 4.5g/L D-glucose, (+)Pyruvate | Thermo Fisher,41965-039 | 500 mL |
| Fetal Bovine Serum (FBS) | Merck, S 0615 | 50 mL |
| Penicillin-Streptomycin solution | Gibco, 15140122 | 5 mL |

Supplementary Table S4. Cell culture medium for HUVEC

| Name | Manufacturer | Amount |
| --- | --- | --- |
| Endothelial Cell Growth Medium | C-22010 | 500 mL |
| Supplemen tMix | C-39215 | 12.2 mL |

Supplementary Table S5. qPCR primer

| Primers | Sequence | Size (bp) | Temp (°C) |
| --- | --- | --- | --- |
| CBS F | CGGCTTCGACTGGGTGTACT | 20 | 64.8 |
| CBS R | GCAGCCTCCCGATTTGG | 17 | 69.5 |
| HIF2α F | CCGCAGTTGTGCTCCTGAA | 19 | 64.8 |
| HIF2α R | ACCTTGGCGGTCTCGTAGCT | 20 | 63.9 |
| ZEB1 F | AAGAATTCAGTGGAGAGAAGCCA | 25 | 64.6 |
| ZEB1 R | CGTTTCTTGAGTTTGGGATT | 22 | 64.3 |


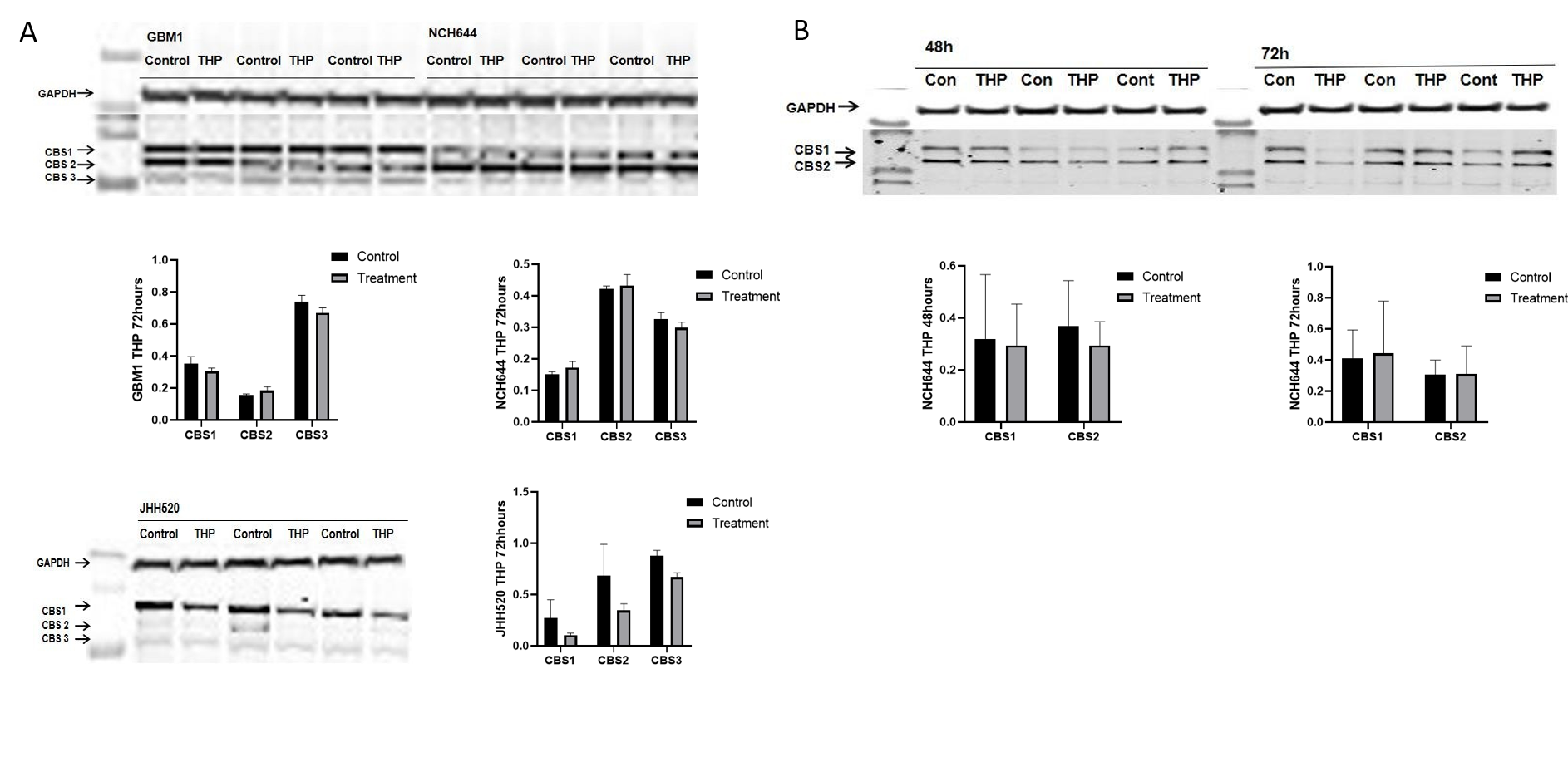


Supplementary Figure S1: CBS-L related protein expression in GBM1, NCH644, JHH520 with or without THP treatment for 72 hours (A). CBS-L related protein expression kinetics in NCH664 with or without THP treatment for 48 or 72 hours (B).
